# Hepatocellular Carcinoma in the Absence of Cirrhosis in a Child With Inactive Chronic Hepatitis B Infection

**DOI:** 10.1097/PG9.0000000000000124

**Published:** 2021-09-23

**Authors:** Charanya Rajan, Fang Kuan Chiou, Khurshid Merchant, Kong Boo Phua

**Affiliations:** From the * Gastroenterology, Hepatology and Nutrition, Department of Paediatrics, Kandang Kerbau Women’s and Children’s Hospital, Singapore; † Department of Pathology and Laboratory Medicine, Kandang Kerbau Women’s and Children’s hospital, Singapore; ‡ Duke-National University of Singapore Medical School, Singapore.

**Keywords:** absence, active hepatitis, chronic hepatitis B, cirrhosis, hepatocellular carcinoma

## Abstract

Chronic hepatitis B infection has been identified as an important risk factor for developing hepatocellular carcinoma (HCC) especially in the presence of hepatitis and liver cirrhosis. However, here we describe an unusual case of a child with chronic hepatitis B infection who developed HCC in the absence of active hepatitis or cirrhosis. This case serves to highlight the importance of regular HCC surveillance for all children with chronic hepatitis B, regardless of presence or absence of hepatitis or cirrhosis.

## INTRODUCTION

Chronic hepatitis B (CHB) infection is an important risk factor for hepatocellular carcinoma (HCC). The pathogenesis is presumably due to chronic hepatocyte inflammation and necrosis causing cirrhosis and nodular regeneration progressing to formation of dysplastic nodules and eventual HCC (1). Patients with hepatitis B e antigen (HBeAg)-negative HBV infection with low HBV DNA and no active hepatitis are considered to have low risk of progression to cirrhosis and HCC (2). We describe an unusual case of a 13-year-old boy with HBeAg-negative chronic HBV infection who developed HCC in the absence of active hepatitis or cirrhosis.

## CASE SUMMARY

The patient was born to a HBV carrier mother. He received HBV immunoglobulin at birth and the full course of HBV vaccine at 1 day, 1 month, 6 months, and 13 months of life. He was lost to follow-up until he was 9 years old when an incidental blood screen showed positive hepatitis B surface antigen. Further evaluation was negative for hepatitis B surface antibody and HBeAg but positive for hepatitis B e antibody with low level of HBV DNA at 1961 copies/mL and a normal liver function test (LFT). He was completely asymptomatic. He was followed up annually with normal LFT and ultrasound imaging of his hepatobiliary system. His alanine transaminase (ALT) level was 14 to 17 U/L and aspartate transaminase (AST) level was 19 to 23 U/L and always remained within normal limits. His platelets level ranged between 197 and 223 × 10^9^/L.

**FIGURE 1.**
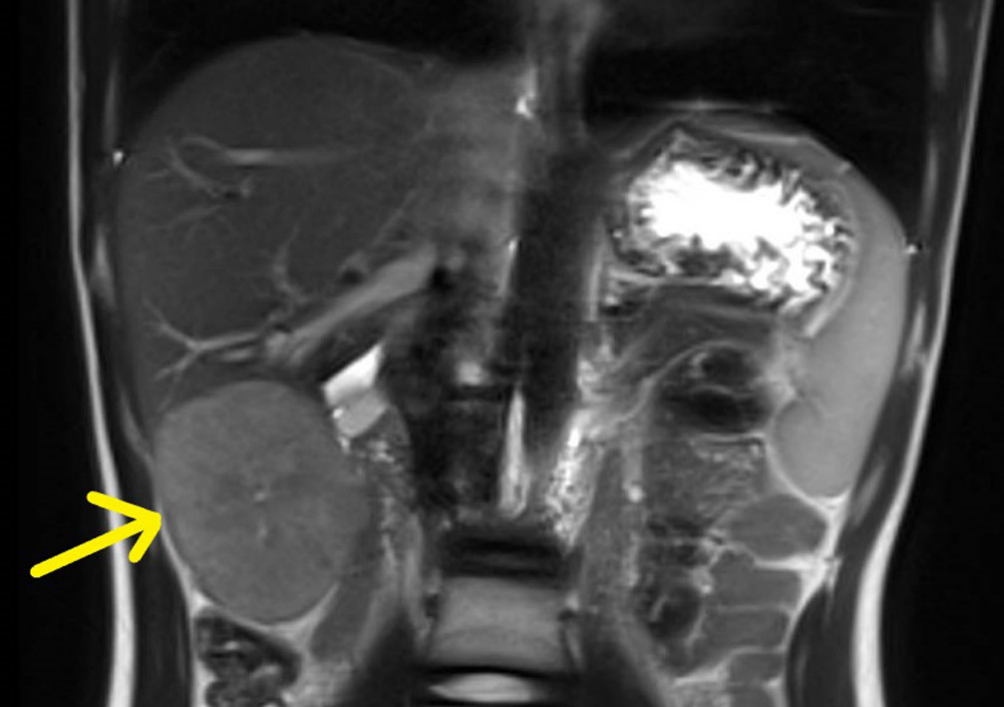
Magnetic resonance imaging of 7.5 by 6.5 by 6 cm well-defined mass in the inferior right hepatic lobe (yellow arrow). The mass does not extend to the liver hilum and does not involve the portal vein or inferior vena cava. Spleen span is normal at 11 cm.

During a routine follow-up at 13 years old, the patient reported left upper-quadrant abdominal pain. Clinical examination revealed isolated hepatomegaly. LFT showed slightly increased levels of ALT and AST of 37 U/L each. HBV DNA load was 6233 copies/mL and the virus was of genotype B, the commonest type in Singapore. Alpha-fetoprotein level was significantly raised at 53 606 µg/L. Magnetic resonance imaging of the hepatobiliary system showed a well-defined mass in the inferior right hepatic lobe (Fig. 1).

**FIGURE 2.**
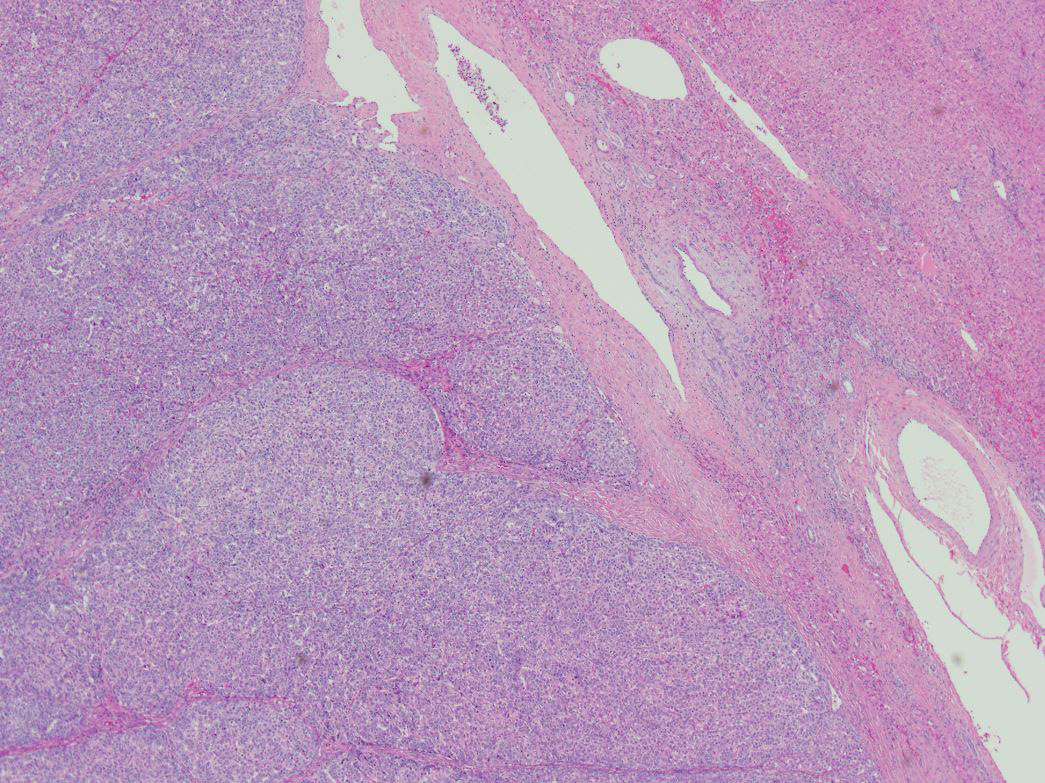
Haematoxylin and eosin stained section, ×4 original magnification, demonstrates well-circumscribed hepatocellular carcinoma featuring solid nodules of pleomorphic tumor and adjacent noncirrhotic hepatic parenchyma.

**FIGURE 3.**
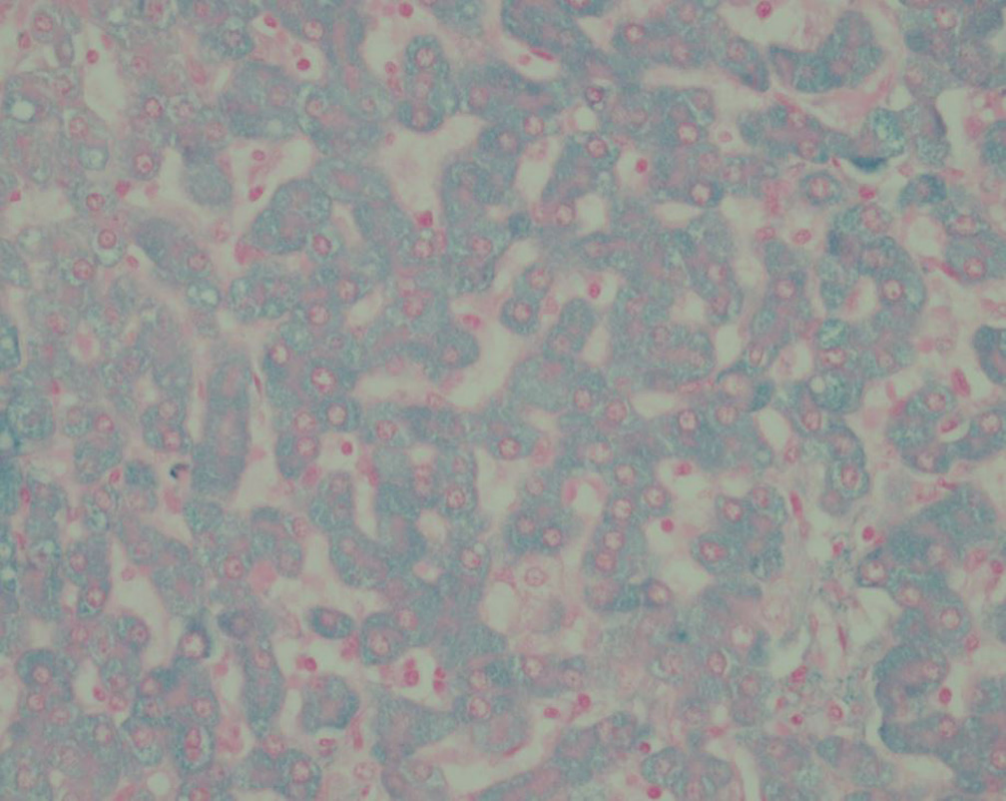
Victoria Blue immunostain, original magnification ×4 highlights blue staining hepatitis B surface material in a pink background in the uninvolved hepatic parenchyma.

The patient underwent complete resection of the tumor together with segment 5 and 6 segmentectomy. Intraoperative findings showed a 7-cm solitary exophytic liver tumor growing from segments 5 and 6. There were no satellite lesions nor any peritoneal or omental nodules. Histopathology was in keeping with grade 3 HCC with no underlying hepatic fibrosis and cirrhosis (Figs. 2, 3). A staging computed tomography of the chest did not show any metastases.

One month following his surgery, the patient was commenced on entecavir. No chemotherapy was initiated. In the 5 years of follow-up since surgery, he has remained symptom-free. His alpha-fetoprotein reduced to undetectable levels 6 months’ postsurgery. His LFT and HBV DNA levels remained normal while on entecavir. Regular ultrasound imaging of his hepatobiliary system have not shown any focal lesions or hepatic cirrhosis. Vibration-controlled transient elastography at the most recent follow-up showed normal liver stiffness (6.5 kPa).

## DISCUSSION

This report describes a rare case of HCC in an HBeAg-negative patient who had low viral load and no biochemical or histologic evidence of ongoing active hepatitis or liver fibrosis/cirrhosis. Typically, patients who are HBeAg-negative patients with minimal necroinflammatory activity and normal ALT, even in the presence of slightly elevated (2000-20 000 IU/mL) HBV DNA, are considered to be at low risk of progression to cirrhosis or HCC (2). On the other hand, HBeAg-negative chronic active hepatitis (with active viral replication and abnormal ALT levels) which affects 10% of pediatric patients may show more aggressive disease progression and increased risk of HCC (3).

The clinical course of a patient with HBV infection is influenced by the age at primary infection, gender of patient, route of transmission, HBV genotype, and environmental factors (4). An elevated serum HBV level of more than 10000 copies/mL at baseline is in itself a strong predictor of development of HCC (4). A Taiwanese study by Hsu et al (5) suggests that early HBeAg seroconversion could also be a risk factor for HCC, where there were reportedly low positive rates of HBeAg and HBcAg coupled with high frequency of liver cirrhosis. Wen et al (6) also reports in a case series on children with chronic HBV who developed HCC that those who seroconverted to HBeAg before the age of 3 years and in the presence of cirrhosis had a significantly higher risk of HCC than those who did not. This is postulated to be due to hepatocyte necrosis preceding HBeAg seroconversion leading to fibrosis and malignant change. Studies have shown that those with genotype B infection have a higher rate of HBeAg clearance and this could contribute to the early seroconversion (7).

In addition, Fujisawa et al (8) had reported 2 children who developed HCC even while being anti-HBe positive, either by spontaneous seroconversion or following interferon therapy, with no known active hepatitis or cirrhosis while Mogul et al (9) reported a series of 8 pediatric patients with HBV-associated HCC of whom only 2 patients had cirrhosis or bridging fibrosis. However, a significant proportion of patients from Mogul et al’s (9) study had missing data on HBe antigen status and HBV DNA. Similarly, in Zhang et al’s (10) study, cirrhosis was only found in 18 of 32 patients with HBV-associated HCC. However, HBe status was available for only 26 of these patients. Nonetheless, what is clear from these published data and further illustrated by our case is that HCC can occur in CHB in the absence of cirrhosis or significant liver dysfunction. Although uncommon, HBV-associated HCC is associated with a dismal prognosis with survival rates between 25% and 50%. While international guidelines recommend 6 to 12 monthly assessment and HCC surveillance for patients with HBe-negative CHB with low viral loads, it could be argued that for at-risk groups such as patients with genotype B, persistent active hepatitis and/or with family history of HCC, HCC surveillance should be performed at closer intervals of 6 months or less.

In conclusion, this case serves to highlight that chronic inflammation and cirrhosis are not prerequisites for development of HCC in children with CHB infection. While hepatitis B e antibody seroconversion is still considered a favorable outcome in CHB, a regular HCC surveillance for all children with CHB, regardless of HBe-antigen status, presence of active hepatitis or stage of fibrosis, is of paramount importance.

## References

[1] Blum HE Moradpour D. Viral pathogenesis of hepatocellular carcinoma. J Gastroenterol Hepatol. 2002;17(suppl 3):S413–S420. 12472973 10.1046/j.1440-1746.17.s3.37.x

[2] European Association for the Study of the Liver. EASL 2017 clinical practice guidelines on the management of hepatitis B virus infection. J Hepatol. 2017;67:370–398. 28427875 10.1016/j.jhep.2017.03.021

[3] European Association for the Study of the Liver. Management of chronic hepatitis B in childhood: ESPGHAN clinical practice guidelines. Consensus of an expert panel on behalf of the European Society of Pediatric Gastroenterology, Hepatology and Nutrition. J Hepatol. 2013;59:814–829. 23707367 10.1016/j.jhep.2013.05.016

[4] Komatsu H Inui A Fujisawa T. Pediatric hepatitis B treatment. Ann Transl Med. 2017;5:37. 28251116 10.21037/atm.2016.11.52 PMC5326647

[5] Hsu HC Wu MZ Chang MH. Childhood hepatocellular carcinoma develops exclusively in hepatitis B surface antigen carriers in three decades in Taiwan. Report of 51 cases strongly associated with rapid development of liver cirrhosis. J Hepatol. 1987;5:260–267. 2828461 10.1016/s0168-8278(87)80030-2

[6] Wen WH Chang MH Hsu HY. The development of hepatocellular carcinoma among prospectively followed children with chronic hepatitis B virus infection. J Pediatr. 2004;144:397–399. 15001956 10.1016/j.jpeds.2003.11.022

[7] Lindh M Hannoun C Dhillon AP. Core promoter mutations and genotypes in relation to viral replication and liver damage in East Asian hepatitis B virus carriers. J Infect Dis. 1999;179:775–782. 10068571 10.1086/314688

[8] Fujisawa T Komatsu H Inui A. Long-term outcome of chronic hepatitis B in adolescents or young adults in follow-up from childhood. J Pediatr Gastroenterol Nutr. 2000;30:201–206. 10697141 10.1097/00005176-200002000-00019

[9] Mogul D Ling S Murray K. Characteristics of Hepatitis B virus –associated hepatocellular carcinoma in children: a multi-center study. J Pediatr Gastroenterol Nutr. 2018;67:437–440. 30063586 10.1097/MPG.0000000000002093

[10] Zhang XF Liu XM Wei T. Clinical characteristics and outcome of hepatocellular carcinoma in children and adolescents. Pediatr Surg Int. 2013;29:763–770. 23794023 10.1007/s00383-013-3334-4

